# Survivorship During Starvation for *Cimex lectularius* L.

**DOI:** 10.3390/insects2020232

**Published:** 2011-05-11

**Authors:** Andrea M. Polanco, Dini M. Miller, Carlyle C. Brewster

**Affiliations:** Department of Entomology, Virginia Tech, 216A Price Hall, Blacksburg, VA 24061, USA; E-Mails: apolanco@vt.edu (A.M.P.); carlyleb@vt.edu (C.C.B.)

**Keywords:** *Cimex lectularius*, bed bug, starvation, resistance, survivorship, pyrethroids

## Abstract

Four bed bug strains (*Cimex lectularius*) with different levels of pyrethroid resistance were evaluated to determine their ability to survive extended periods of starvation. First instar bed bugs of all strains were the most vulnerable to starvation (13.8–36.3 days mean survival time). Fifth instars and adults survived the longest during starvation (41.5–142.6 days). Significant differences in survivorship during starvation were observed between resistant and susceptible strains of bed bugs. Overall, all immature and adult stages of the resistant bed bug strains had significantly shorter survival times than those of the susceptible strains (*P* < 0.05).

## 1. Introduction

The relationship between humans and bed bugs (*Cimex lectularius*) has been well documented throughout history [1]; however, the biology and ecology of this once severe pest has not been actively studied for over 50 years. This paucity of bed bug research was largely due to the bed bug's near elimination from developed nations after the widespread use of DDT and other insecticides [1]. Most of the literature regarding the biology and ecology of the common bed bug is, therefore, potentially outdated [2]. For example, bed bug resistance to insecticides either did not exist or was unknown when biological studies were conducted in the 1940s. Therefore, it is very likely that modern bed bug populations (particularly resistant populations) are at least somewhat different from the bed bug populations described by Johnson [2] or Omori [3], with respect to their fitness, and specifically their survivorship.

In recent years there has been a significant increase in bed bug infestations throughout the U.S. [4,5]. The growing number of infestations in the U.S. is an indicator of a more widespread increase across the globe [6]. The recent proliferation of bed bugs has been attributed to immigration and emigration [4,7], an increase in international travel (including vacationing) [8], and, most importantly, bed bug resistance to pyrethroid insecticides [9].

The majority of insecticides currently used for bed bug control come from a single class, the pyrethroids. Repeated pyrethroid applications over time, however, have resulted in bed bug populations becoming resistant [9–12]. Moore and Miller [10] reported resistance to deltamethrin in a field strain of bed bugs collected in Virginia. Romero *et al.* [11] also reported resistance to deltamethrin and lambda-cyhalothrin in populations collected in California, Florida, Kentucky, Ohio, and Virginia.

Pyrethroid-resistant bed bugs have been documented to have at least two types of physiological resistance: *kdr*-type mutations [12] and enhanced production of detoxification enzymes [13]. Enhanced production of detoxification enzymes may result in a reduction in fitness for some populations. Resistant insects that invest energy in insecticide detoxification have less energy to put into reproduction and survivorship [14–16]. However, the detoxification of insecticides can interfere with the natural physiology of the insect by changing their enzyme activity and function, and thus reduce the insect's adult life span [15]. Alternatively, the wasteful overproduction of an enzyme when there is no insecticide to detoxify may also reduce the insect's longevity [17]. This reduction in life span due to insecticide resistance has been documented in several insect families, including Aphididae [18], Tortricidae [19], Culicidae [20], and Muscidae [21–23]. Even when no insecticide is present, resistant strains are found to be at a selective disadvantage because the alleles that confer resistance have negative effects on the resistant individuals [24].

Environmental stressors such as high temperatures or starvation also have negative impacts on bed bug survivorship [3]. However, bed bugs are reported to be particularly adept at enduring long periods of starvation [25]. Omori [3] reported that starved adult female bed bugs could survive an average of 277 days at 18 °C, and as long as 425 days at 10 °C. Because bed bug response to starvation has not been evaluated for many decades, it is possible that current resistant populations may respond differently to extended periods of starvation than those evaluated by Omori in 1941.

The purpose of this study was to quantify the survivorship of all bed bug life stages (first-fifth instars and adults) during an extended period of starvation. In this study, four bed bug strains (two pyrethroid-susceptible and two resistant strains) were evaluated to determine whether there were differences in survivorship among the populations.

## 2. Materials and Methods

### 2.1. Bed Bug Rearing

A susceptible laboratory strain of bed bugs (HS) was acquired in February 2005 from Harold Harlan (National Pest Management Association, Fairfax, VA, USA). Dr. Harlan maintained this population of bed bugs, feeding them on himself since 1973. The population was originally collected from Fort Dix, NJ, USA. Two field strains were also collected in 2008. The Richmond strain (RR) was collected from apartments in Richmond, VA, USA in September and the Epic Center strain (ER) was collected in June from a hotel room in Cincinnati, OH, USA. Finally, the British field strain (BS) was collected in September 2006 from an apartment in London, England and a portion of this population was sent to the Dodson Urban Pest Management Laboratory in 2008. All strains were evaluated to determine their susceptibility to pyrethroid products labeled for indoor use in the United States (active ingredients: lambda-cyhalothrin (0.03%), bifenthrin (0.02%), deltamethrin (0.06%), permethrin (0.5%)). Product evaluations were conducted by confining bed bugs on dried insecticide residues to determine LT50 values [26] as described by Miller and Moore [10]. Both HS and BS were determined to be pyrethroid- susceptible strains, while RR and ER were found to be highly resistant to all products tested. Table 1 presents the results of the LT_50_ analysis to determine bed bug strain susceptibility to deltamethrin (0.06%; Suspend SC, Bayer CropScience, Research Triangle Park, NC, USA).

The bed bug colonies were reared in plastic jars covered at one end with a cloth mesh. Two pieces of cardboard were placed inside of the jars so that bed bugs could crawl up the cardboard and feed through the cloth mesh from an artificial feeder. Circulating hot water was used to maintain a diet of chicken blood (formulated with sodium citrate as an anti-coagulant) at 35.5 °C in the artificial feeder. Bed bugs were fed once a week on the chicken blood. Between feedings, bed bugs were stored in an environmental chamber at 26.1–26.5 °C, 68.9% RH, and photoperiod of 12:12 h, L:D. These conditions closely approximated the conditions at which Johnson [2] evaluated bed bug populations for maximum average fecundity and longevity. All bed bug colonies were maintained in the Dodson Urban Pest Management Laboratory (Virginia Tech, Blacksburg, VA, USA).

### 2.2. Evaluation of Survivorship during Starvation

For this experiment two pyrethroid susceptible strains, HS and BS, and two resistant strains, RR and ER, were selected for evaluation. The individual bed bugs were observed under the microscope to determine their developmental stage [1]. Recently molted individuals from each developmental stage (first–fifth instars and adults) were randomly selected from each strain. Five groups of 10 individuals from the same stage were placed in Petri dishes (Fisher brand 60 × 15 mm) containing a single filter paper (Whatman 42.5 mm). A total of 50 individuals from each life stage were evaluated. Adult males and females were also placed in separate Petri dishes to prevent copulation. No blood meals were offered to any of the bed bugs after they were place inside the Petri dishes. The Petri dishes containing the bed bugs were checked daily for mortality, and the data were recorded until all individuals had died.

### 2.3. Statistical Analysis

We calculated the mean survival time for the 10 randomly selected individuals in each Petri dish. This resulted in five values of mean survival times at each developmental stage for each of the four strains. Differences in within-stage survival times among bed bug strains and within-strain survival times among developmental stages were determined using a randomization test within a one-way analysis of variance (ANOVA) [28,29]. The randomization ANOVA used here is a nonparametric alternative to conventional ANOVA and is particularly useful for analysis of unreplicated data and N-of-1 experiments [28–30]. As a first step in the analysis, we calculated a test statistic for the original data. The test statistic of choice was the observed *F*-value (*F*_obs_) from the ANOVA [28,29]. We then randomly permuted and reassigned the mean survival values among the levels of the factor (bed bug strain or developmental stage) to maintain the original number of five values within each factor level. Following this, we reran the ANOVA to calculate a new *F*-value. Because it was impractical and unnecessary to run all of the possible permutations of our data for each analysis, we elected to use 4999 permutations [29] to develop the distribution of *F*-values. The *P*-value for each analysis was determined by the proportion of the 5000 *F*-values (i.e., 4,999 permuted *F*-values + *F_obs_*) that were greater than *F_obs_*. Statistical significance was determined by a *P*-value ≤ α = 0.05. Following the randomization ANOVA, we calculated both the 83% and 95% bootstrap confidence intervals [29] for the mean survival time at each factor level. Significant differences in the mean survival times among factor levels were determined by failure of the 83% confidence intervals to overlap [31,32]. All of the analyses were carried out using MATLAB R2010b 7.11 with Statistical Toolbox (The MathWorks Inc., Natick, MA, USA).

## 3. Results

Analysis of bed bug survival times with the randomization ANOVA showed that there were significant differences in the within-stage survival times among bed bug strains (*F_obs_* (_3_,_16_) = 5.71–20.96, *p* < 0.05) and in the within-strain survival times among developmental stages (*F_obs_* (_6_,_28_) = 4.52–12.99, *p* < 0.05) (Table 2). As expected, the first instars of all strains were more susceptible to starvation than the larger nymphs and adult stages. First instars of all strains survived an average of 13.8-36.3 days of starvation (Table 2). Second instars of all strains were significantly more resistant to starvation than the first instars within their own strain. Second instar mortality occurred between 22.5 and 74.2 days. In general, once the bed bugs were in the third or fourth instar their starvation survival times were similar to those of older instars and adults; however, there were differences observed in developmental stage survival times among the strains.

Mean survival time for first instars of the HS strain was significantly less (36.3 days) than that of all other instars and adults (Table 2). Second instar mean survival time was greater (56.3 days) than that of the first instars but less than the mean survival time of all other instars. Survival times of the third, fourth, and fifth instars, and adult bed bugs were not significantly different.

Young nymphs (first and second instars) of the BS strain were also the most susceptible to starvation (Table 2). However, the older nymphs (third–fifth instars) were significantly less susceptible to starvation than the adults. The later instars of the BS strain survived between 116.4–142.6 days without a blood meal while the adults only survived between 73.9–92.9 days. In fact, the middle instars of the BS strain were by far the least susceptible to starvation of all the bed bugs tested.

The survival time for the ER nymphs increased significantly with each instar (Table 2). The fifth instar had the greatest ability to resist starvation surviving significantly longer than any other instar and the adults.

The RR strain was the most susceptible to starvation of all the strains tested, with no development stage (or individual) expected to survive more than 51 days. Similar to the other strains, the first instars of the RR strain were the most vulnerable stage, surviving on average only 13.8 days. Second and third instars survived significantly longer than first instars but did not live as long as fourth instars. There were no significant differences in the life length between starved fifth instars and adults in the RR strain, with each surviving an average of ∼43 days.

Overall, mean survival time during starvation differed significantly among the strains. What is most obvious is that both of the resistant bed bug strains (RR and ER) were significantly more vulnerable to mortality during starvation than the susceptible strain of bed bugs at all developmental stages (Table 2).

## 4. Discussion

Laboratory studies characterizing life table parameters for laboratory and field strains of bed bugs determined that bed bug eggs hatch within 6–9 days of being produced. If these newly hatched first instars receive a blood meal, they will typically molt to the next instar within 5 days. Molting within 5 days of a blood meal is typical of all nymphal stages provided the individuals are allowed to feed to repletion [27]. Thus, the instar itself is a means of quantifying the number of complete blood meals a particular bed bug has taken. For example, a third instar bed bug has taken two complete blood meals, one as a first instar, and another as a second instar. In this study, to accurately measure the life length of a particular instar during starvation, it was very important to provide the blood meal during the previous instar (except for the first instar), wait for the individual insect to molt, and then starve it during the instar that was to be measured.

Because the first instar bed bug was the only developmental stage that did not receive a blood meal, it was not surprising that starved individuals in this stage, overall, had the shortest life length of all instars and developmental stages tested. Other studies reported the first instar as the most susceptible to starvation [1,2,33]. One explanation for high mortality in first instar bed bugs is undoubtedly moisture loss. Recently hatched bed bugs lose moisture easily because they have a larger surface area compared to their volume [1]. If they are unable to feed there are no means of replenishing this moisture. In our study, first instar bed bugs survived 11.5–37.7 days (depending upon the strain). Kemper [33] reported starved first instars surviving 83.7 days. However, the Kemper first instar bed bugs were held at a lower temperature (22 °C and 40–45% RH). Several studies have documented that starved bed bugs survive longer at lower temperatures [3,34,35]. It should also be mentioned that from the Kemper [33] study it is unclear whether the first instar bed bugs were ever fed, or if they were fed once and survivorship during starvation quantified after they had molted to the second instar.

While we observed that second and third instars did survive longer than first instars in all strains, the field strains did not live as long as other strains, as previously reported in the literature. Omori [3] found that at 27 °C and 70–75% RH, third instar bed bugs survived on average 71.2 days after the last blood meal. However, we found that third instar bed bugs from the field strains, ER and RR, survived no longer than ∼38 days on average after their last blood meal when held at a similar temperature.

The current study also found that the mean survival times after starvation of virgin adult male and female bed bugs in each strain were similar (43.7–92.6 days for adult females and 42.9–99.4 days for adult males). Interestingly, these results also conflict with those of Omori [3], who stated that virgin adult females lived longer than males during starvation (43.4 days for males and 86.7 days for females). Johnson [2] and Kemper [33] found that with mated adults, males typically outlived females, except at very low temperatures. Yet, both authors reported that virgin females lived longer than virgin males. However, it is difficult to determine from the text whether the authors meant that virgin females lived longer at low temperatures or at all temperatures.

*Cimex lectularius* survivorship under different conditions has been studied by many researchers [2,3,33,35,36]. Not surprisingly, each study provided different results regarding how long a bed bug can live without a blood meal. The Kemper [33] study was conducted at 22 °C and 40–45% RH, and reported first and second instars surviving for 83.7 days and adults surviving for 130.6–142.6 days during starvation. Bacot [35] reported keeping starved bed bugs from all developmental stages in an outhouse for 18 months. Gunn [36] reported adult bed bugs living for more than three years with sporadic feedings (approximately 23 feedings over that period of time). However, the temperature and relative humidity for this experiment were not stated. Johnson [37] also reported a female bed bug living for more than 580 days after the last blood meal. The results of the current study were quite different from those reported prior to 1950. In fact, susceptible bed bug strains, which are rarely found in the field, had the longest survival times, and yet did not live as long as 130 days (on average). The maximum life span observed in this study was recorded for a single fifth instar bed bug from the BS strain, which lived ∼143 days without a blood meal. The field strains of bed bugs did not live beyond 80 days.

The fact that we found significant differences in the survivorship for almost every developmental stage between strains suggests that each of the strains is physiologically different. There is no question that the Harlan strain has been through a genetic bottle neck after being in the laboratory for over 36 years. Therefore, we would expect that the response of the HS bed bugs would be significantly different from that of the field stains. The BS strain has only been in the laboratory since 2006. However, the BS strain would also be considered susceptible based on the resistance ratio criteria established by Cochran [26]. While the BS strain had formerly been considered a resistant strain [38], its physiological resistance has obviously declined while it has been in the laboratory, indicating that like the Harlan strain, it has become a more homogeneous culture. Perhaps life in the laboratory has selected for those individuals that can withstand long periods of starvation. However, given that laboratory strains are typically pampered with regular blood meals to maximize reproduction, selection for individuals able to withstand long period of starvation seems unlikely.

Because the life expectancy of the two field-collected strains was determined to be significantly lower than either of the susceptible laboratory strains leads us to speculate that insecticide resistance may actually play a role in bed bug survivorship during starvation. Several populations of insecticide resistant insects have been documented to have reduced survivorship compared to susceptible populations [24]. Groeters *et al.* [39] reported that resistant populations of diamondback moth (*Plutella xylostella*) had shorter adult life spans than susceptible populations. Sarita *et al.* [20] found a significant reduction in adult longevity in a strain of *Aedes aegypti* resistant to deltamethrin when compared with susceptible populations. Rosenheim and Tabashnik [40] used a model to simulate and measure fitness for a hypothetical insecticide resistant insect. The model predicted a significant loss of fitness in resistant populations, even in the absence of the insecticide. While we cannot make specific predictions about the survivorship of different bed bug populations collected from the field in relation to insecticide resistance, subsequent studies may determine that there is a correlation between the level of resistance of a population and its ability to survive environmental stressors (e.g., periods of starvation).

Our study may help to update some internet-accessible information regarding bed bug survivorship during periods of starvation. Currently, it is very common to find bed bug information sites stating that bed bug populations can live for a year or more without a blood meal. For instance, the BadBedBug.com states “bed bug adults can survive more than 12 months without feeding” [41]. BedBugs.org states: “adult bed bugs can survive for up to 7 months without a blood meal and have been known to live in empty buildings for up to one year” [42]. Even fact sheets from universities report long periods of bed bugs survival: “immature bed bugs may live for several months without feeding while adults may survive as long as one year” [43]. However, these sites give no references as to the origin of the information. No doubt these references to long periods of survivorship during starvation in bed bugs originated from the older studies conducted by Johnson [37], Omori [3] and Busvine [34]. However, these websites neglect to mention that the data were from studies where insecticide resistance was either not quantified or unknown, and that the long survival times were most likely recorded for bed bugs held at temperatures below at 11 °C [3]. The results of this study indicate that the bed bugs strains currently infesting homes and apartments within the United States do not survive starvation nearly as long as previously reported.

## 5. Conclusions

Our studies indicated that first instar bed bugs were the most vulnerable to mortality during starvation, surviving only one month or less. While second instars survived significantly longer (1.0-2.5 months) than first instars, their survival period during starvation was still significantly less than that of older instars or adults (up to 4 months depending on the strain). Fifth instar and adult bed bugs were the least susceptible to mortality during starvation; however no individual survived more than 135 days. This result was surprising because most popular references state that adult bed bugs are capable of surviving for more than a year without feeding. Significant differences were observed in length of survival during starvation between all strains; however, the greatest differences were observed between those strains that were insecticide resistant and those that were susceptible. Resistant strains were found to be much more vulnerable to mortality during starvation than susceptible bed bugs. Overall, each resistant developmental stage (instar or adult) survived only one half to one third as long as the same developmental stage in the susceptible strain.

## Figures and Tables

**Table 1:** LT_50_ values (% hours) calculated for adult bed bugs (50% male: 50% female) confined on hardboard panels treated with residual insecticide [27]. Field samples were run same day with Harlan (HS) samples for comparison.

**Bed Bug Strain**	**Test Date**	**Treatment**	**LT_50_ (hours)**	**95% CLs**	**Resistance Ratios**
Harlan (HS)	February 2009	Suspend SC	0.8	0.72–0.94	
Richmond (RR)		Suspend SC	320.2	304.4–338.2	390.5
Harlan (HS)	July 2010	Suspend SC	0.5	0.47–0.54	
British (BS)		Suspend SC	1.3	1.30–1.37	2.6
Harlan (HS)	August 2009	Suspend SC	1.1	1.06–1.19	
Epic Center (ER)		Suspend SC	>384	---	>340

**Table 2:** Mean (95% CI) survival times (days) during starvation for immature and adult stages of two pyrethroid-susceptible (HS and BS) and two pyrethroid-resistant (RR and ER) bed bug strains (reared at 26.1–26.5 °C, 68.9% RH, photoperiod 12:12 hour L:D).

**Bed Bug Strain**	**Mean Survival Time (days) during Starvation (95% CI)**						

	**Instar**					**Adult**	

	**1st**	**2nd**	**3rd**	**4th**	**5th**	**Female**	**Male**
Harlan (HS)	36.3 a, A (35.4–37.7)	56.3 b, A (53.2–59.1)	90.1 c, A (85.9–100.1)	95.8 c, A (91.5–105.8)	99.3 c, A (95.5–107.7)	92.6 c, A (88.9–99.1)	99.4 c, A (94.9–106.3)
British (BS)	28.6 a, B (27.3–31.6)	68.3 b, B (65.0–74.2)	118.1 c, B (116.4–123.0)	124.2 d, B (120.4–131.9)	127.9 d, B (118.1–142.6)	74.5 e, B (73.9–79.5)	82.6 f, B (78.4–92.9)
Epic Center (ER)	15.0 a, C (10.9–19.8)	27.9 b, C (22.5–32.4)	38.3 c, C (36.2–44.4)	61.0 d, C (55.4–71.7)	73.2 e, C (69.9–79.5)	62.5 d, B (55.2–76.3)	60.3 d, C (59.9–63.9)
Richmond (RR)	13.8 a, C (11.5–16.4)	27.8 b, C (26.5–30.3)	28.2 b, D (25.1–32.1)	33.4 c, D (30.6–38.2)	43.7 d, D (41.5–48.5)	43.7 d, D (39.0–50.9)	42.9 d, D (42.1–47.2)

* Significant differences in the mean survival times among factor levels were determined by the non-overlapping 83% confidence intervals. Means across each row (within strain) followed by the same lowercase letter are not significantly different. Means within each column (between strains) followed by the same uppercase letter are not significantly different.
